# Antioxidant treatment ameliorates prefrontal hypomyelination and cognitive deficits in a rat model of schizophrenia

**DOI:** 10.1038/s41386-021-00964-0

**Published:** 2021-02-09

**Authors:** D. A. Maas, V. D. Eijsink, J. A. van Hulten, R. Panic, P. De Weerd, J. R. Homberg, A. Vallès, B. Nait-Oumesmar, G. J. M. Martens

**Affiliations:** 1 Dept of Molecular Animal Physiology, Donders Centre for Neuroscience, Radboud University, Nijmegen, The Netherlands; 2 Sorbonne Université, Paris Brain Institute-ICM, Inserm U1127, CNRS UMR 7225, Hôpital Pitié-Salpêtrière, Paris, France; 3 Dept of Cognitive Neuroscience, Donders Centre for Medical Neuroscience, Radboud University Medical Center, Nijmegen, The Netherlands; 4 Dept of Neurocognition, Faculty of Psychology and Neurosciences, Maastricht University, Maastricht, The Netherlands; 5 Maastricht Centre of Systems Biology, Faculty of Science and Engineering, Maastricht University, Maastricht, The Netherlands

**Keywords:** Neuroscience, Schizophrenia

## Abstract

Cognitive dysfunction in schizophrenia (SZ) is thought to arise from neurodevelopmental abnormalities that include interneuron hypomyelination in the prefrontal cortex (PFC). Here we report that RNA-sequencing of the medial (m)PFC of the APO-SUS rat model with SZ-relevant cognitive inflexibility revealed antioxidant metabolism as the most-enriched differentially expressed pathway. Antioxidant-related gene expression was altered throughout postnatal development and preceded hypomyelination. Furthermore, reduced glutathione levels and increased mitochondria numbers were observed in the mPFC. Strikingly, chronic treatment with the glutathione precursor N-acetylcysteine (NAC) from postnatal days 5–90 restored not only antioxidant-related mRNA expression and mitochondria numbers, but also myelin-related mRNA expression and mPFC-dependent cognitive dysfunction, while blood glutathione levels remained unaffected. The promyelinating effect of NAC was at least partly due to a positive effect on oligodendrocyte lineage progression. Together, our findings highlight that oxidative stress may contribute to cognitive symptoms in the APO-SUS rat model of SZ and encourage antioxidant therapy in early phases of SZ.

## Introduction

Schizophrenia (SZ) is a severe neurodevelopmental disorder that includes cognitive symptoms arising from the prefrontal cortex (PFC). The neurobiological basis of the cognitive symptoms of SZ remains elusive. Oxidative stress, defined as an imbalance between the production of reactive oxygen species (ROS) and the clearance of ROS by antioxidants, has been suggested to play a key pathological role [1]. In SZ patients, oxidative stress has been reported in the blood and cerebral spinal fluid as well as the medial PFC (mPFC) and is assumed to be caused by decreased glutathione antioxidant levels [2]. Glutathione levels are reduced in blood plasma, cerebral spinal fluid, PFC and postmortem brain regions of SZ patients, and are independent of disease stage or medication use [3]. Notably, decreased levels of antioxidants are correlated with impaired PFC-dependent cognitive ability in first-episode SZ [4]. Glutathione antioxidant levels are also associated with white-matter integrity in the PFC [5]. In SZ, white-matter abnormalities are presumably caused by defective myelination, correlate with cognitive symptoms [6], occur already before SZ onset and aggravate further following transition to psychosis [7]. As decreased levels of glutathione are associated with both white-matter abnormalities and interneuron maturation defects in the PFC of SZ patients, the question remains whether oxidative stress is a key player and possibly a causative factor in the development of mPFC interneuron hypomyelination in SZ.

To explore this question, we used a well-characterized, idiopathic rat model with SZ-relevant features, namely the apomorphine-susceptible (APO-SUS) rat along with its phenotypic counterpart, the apomorphine-unsusceptible (APO-UNSUS) rat. APO-SUS rats do not require genetic or pharmacological manipulation to display SZ-relevant behavioral characteristics, such as reduced prepulse inhibition, increased exploratory behavior, dopamine-induced stereotypic behavior [8–10], reduced latent inhibition and sucrose preference [11, 12] and memory deficits [13]. Furthermore, APO-SUS rats display several neurobiological features similar to those found in SZ [14, 15], including mPFC parvalbumin interneuron hypomyelination [16].

Here, we report that RNA-sequencing (RNA-seq) analysis of the mPFC of APO-SUS and APO-UNSUS rats revealed a central role for glutathione antioxidant metabolism. Intriguingly, treatment with the glutathione precursor N-acetylcysteine (NAC) restored not only glutathione metabolism, but also improved mPFC hypomyelination and cognitive functioning of APO-SUS rats.

## Materials and methods

### Animal model

Generation of the APO-SUS and APO-UNSUS rat lines has been described in detail elsewhere [11, 17]. Briefly, we selectively bred rats from an outbred Nijmegen Wistar rat population that displayed stereotyped behavior upon injection of apomorphine (APO-SUS rats). The same selective breeding was performed with the rats that showed a weak apomorphine-induced stereotypy (APO-UNSUS rats). Apomorphine injection and behavioral selection were only performed with the first 15 generations of APO-SUS and APO-UNSUS rats. In the subsequent breedings, APO-SUS rats displayed SZ-relevant features without this pharmacological treatment. In this study, naive male APO-SUS and APO-UNSUS rats from the 32th–44rd generation were used. Rats were housed in pairs in a temperature- and humidity-controlled room with a 12-h light-dark cycle (lights on at 7.00 a.m.) and ad libitum access to water and standard laboratory chow (V1534-703, SSNIFF, Germany), unless otherwise indicated. Cages with APO-SUS and APO-UNSUS rats were alternately situated to minimize effects of lighting and airflow. Rats from the same litter were divided over different experiments (for example, behavioral testing, immunohistochemistry, electron microscopy or qPCR) to avoid batch effects and sample size was based on previous studies [12, 15, 16]. Researchers were blinded during all ex vivo experiments and data analyses. During behavioral experiments researchers were not blinded as APO-SUS and APO-UNSUS rats are readily recognizable by their different attitude towards humans. Therefore, behavioral experiments were conducted in operant conditioning chambers that were placed in light and sound-attenuating cubicles to minimize the effect of the researcher on the performance of the rats. All animal experiments were approved by the Central Committee on Animal Experiments (Centrale Commissie Dierproeven, CCD, The Hague, The Netherlands) and were conducted in accordance with the Directive 2010/63/EU.

### Micropunching

For micropunching, naive postnatal day (P)21 (±1 day), P60 (±1 day), P90 (±1 day), P120 (±1 day) and P365 (±14 days) APO-SUS and APO-UNSUS rats were sacrificed by direct decapitation and brains were isolated, frozen on dry ice and stored at −80 °C. Micropunching was performed in a cryostat (Leica) at −15 °C and the Paxinos and Watson rat brain atlas was taken as a reference to aid the dissection. mPFC was collected with a 1.20 or 2.00-mm punch needle (Harris) from 300 µm coronal sections at Bregma 4.00–2.20. Dissected tissues were immediately frozen on dry ice and stored at −80 °C until further analysis.

### RNA isolation

RNA for RNA-seq analysis was extracted from P365 APO-SUS and APO-UNSUS mPFC with a Nucleospin RNA II kit following the instructions of the manufacturer (Macherey-Nagel, Dueren, Germany). For all other RNA isolations, tissues were homogenized using Trizol reagent (Sigma) and stainless-steel beads in a Tissue Lyser (Qiagen), followed by chloroform extraction and RNA precipitation with isopropanol in the presence of 20 μg of glycogen (Fermentas). Pellets were washed twice with 75% ice-cold ethanol and dissolved in MilliQ H_2_O. RNA concentration and purity were measured using a DS-11 spectrophotometer (Denovix). RNA was kept at −80 °C until further analysis.

### RNA-seq and data analysis

For RNA-seq analysis, total RNA quality of the samples was assayed using Agilent 2100 Bioanalyser (Applied Biosystems). Total RNA concentration was estimated by Qubit Fluorometer (Invitrogen). RNA samples (all with an RNA integrity number RIN ≥ 8.60) prepared from P365 APO-SUS (*n* = 4, pooled) and APO-UNSUS (*n* = 4, pooled) mPFC were analyzed by RNA-seq at the Hudson Alpha Institute for Biotechnology (Huntsville, AL, USA). RNA-seq libraries were formed from ~850 ng total RNA of each sample. RNA-seq was performed using paired end sequencing on Illumina HiSeq (Illumina), at 100 base pairs, generating over 30 million paired reads. RNAseq FASTQ files were analyzed using GeneSifter software (VizX Labs). Transcript abundance was calculated by estimating the reads per kilobase of exon per million mapped reads (RPKM) and normalization to the number of mapped reads was used for comparison. The mRNAs differentially expressed in the APO-SUS versus APO-UNSUS mPFC and meeting our two predefined criteria (fold change ≥1.2; Likelihood Ratio test corrected *p* value <0.05) were used as input data for analysis with the Ingenuity Pathway Analysis (IPA) software package (Qiagen) to identify overrepresented biological pathways with a focus on ‘Canonical Pathways’.

### Quantitative real-time PCR

For quantitative real-time PCR (qPCR) analysis, RNA samples were treated with DNase I (Fermentas) and cDNA was synthesized using the Revert Aid H-minus first strand cDNA synthesis kit (Thermo Scientific). cDNA was subsequently diluted 1:20 in MilliQ H_2_O and stored at −20 °C until qPCR analysis. qPCR samples were pipetted using a robot (Corbett Robotics) and contained 2.0 μL diluted cDNA, 0.8 μL 5 μM forward primer, 0.8 μL 5 μM reverse primer, 5 μL SybrGreen mix (Roche) and 1.8 μL MilliQ H_2_O. qPCR was performed with a Rotor Gene 6000 Series (Corbett Life Sciences) using a 3-step paradigm with a fixed gain of 8. Fifty cycling steps of 95, 60, and 72 °C were applied, and fluorescence was acquired after each cycling step. Primers were designed with NCBI Primer-Blast or Primer Express 2.0 and synthesized by Sigma (for primer pair sequences, see Supplementary Table 1). Melting temperature was used to check whether a single PCR product was generated and the take off and amplification values of the housekeeping genes (*Ywhaz, B-actin, Ppia* and *Gapdh*) were used to determine the normalization factor with GeNorm [18] after which normalized mRNA expression levels were calculated. For analysis of the developmental time course of mRNA expression, P0, P7, P14, and P21 (±1 day) APO-SUS and APO-UNSUS rats were sacrificed by direct decapitation, brains were immediately removed, and mPFCs were freshly dissected, frozen on dry ice and stored at −80 °C for subsequent RNA extraction and qPCR analysis. Furthermore, for the developmental time course analysis, P21 (±1 day), P28 (±1 day), P90 (±1 day), and P365 (±14 days) APO-SUS and APO-UNSUS brains were removed immediately following decapitation, frozen on dry ice and stored at −80 °C for subsequent micropunching, RNA extraction and qPCR analysis (for *n* numbers before and after outlier exclusion see Supplementary Tables 2, 3 and 4).

### Glutathione assay

Levels of the natural antioxidant glutathione were determined in naive P90 APO-SUS (*n* = 8) and APO-UNSUS rats (*n* = 8; *n* = 7 after removing significant outlier) by a kinetic glutathione assay (CS0260 Sigma) that measured the level of total glutathione (oxidized and reduced glutathione). The assay was performed according to the instructions of the manufacturer and glutathione levels were normalized by protein content determination through a bicinchoninic acid (BCA) assay (23225; Thermo Fisher).

### Electron microscopy

For electron microscopy (EM), naive P90 APO-SUS (±1 day; *n* = 3) and naive P90 APO-UNSUS (±1 day; *n* = 3) rats were perfused with 2% PFA/2% glutaraldehyde and perfused brains were removed, postfixed overnight in 2% PFA/2% glutaraldehyde and stored at 4 °C in PBS/0.01% azide until further processing. Sagittal sections of 100 μm were collected using a vibratome (Leica), fixed with 2% osmium tetroxide and contrast was obtained with 5% uranyl acetate. Following ethanol dehydration, sections were embedded in epon resin and ultrathin (70–100 nm) sections were obtained with an ultramicrotome (Leica). Sections were contrasted using lead citrate and 40 non-overlapping ×26,000 images were obtained in infralimbic (IL) or prelimbic (PL) subregions of the mPFC. Myelinated axons were counted in all 40 images of IL region and the G-ratio and axon caliber of all myelinated axons perpendicular to the field of view were measured in FIJI. We analysed myelination in the IL because the myelination difference between APO-SUS and APO-UNSUS rats is largest in this mPFC subregion [16]. The percentage of mitochondrial surface was calculated using 49 equally (200 µm) spaced crosses superimposed over 20 randomly picked IL and 20 randomly picked PL images. The percentage of crosses that touched a mitochondrion over the total number of crosses was calculated. As the percentage mitochondrial surface was lower in both the APO-SUS IL and PL subregions, we performed further mitochondrial analyses on the PL subregion only. In the PL subregion, we measured the size of each mitochondrion that touched a cross as well as the exact number of mitochondria.

### NAC treatment

APO-SUS and APO-UNSUS rats were treated between P5-P40 (±1 day) with 0.9 g of NAC (A7250 Sigma) per liter and from P40-P90 (±1 day) with 2 g of NAC per liter in the drinking water. NAC treatment regimen was based on Cabungcal et al. [19] and Jallouli et al. [20]. As high concentrations of NAC are lethal at young ages, we started with a low dose (0.9 g of NAC per liter) that has successfully been used in pups before [19]. We increased the dose during young adulthood to 2 g of NAC per liter as this dose has been used before in adult rats to reduce oxidative stress for long periods of time [20]. Using 5 M NaOH, the pH was adjusted to 8.6, equivalent to the pH of control drinking water, and NAC as well as control drinking water was offered in dark drinking bottles to prevent NAC oxidation by light. Both NAC-laced and control drinking water were refreshed three times per week. Animals treated with NAC-laced and control drinking water were either sacrificed at P90 (±1 day) for electron microscopy (APO-SUS, *n* = 6; APO-SUS + NAC, *n* = 5; APO-UNSUS, *n* = 5; APO-UNSUS + NAC, *n* = 2) or immunofluorescent analyses (APO-SUS, *n* = 5 (*n* = 4 after removing significant outlier); APO-SUS + NAC, *n* = 6; APO-UNSUS, *n* = 7; APO-UNSUS + NAC, *n* = 10), or were subjected to behavioral testing between P60 and P90 (±6 days; APO-SUS, *n* = 30; APO-SUS + NAC, *n* = 10; APO-UNSUS, *n* = 28; APO-UNSUS + NAC, *n* = 10) and sacrificed at P90 for glutathione assays (APO-SUS, *n* = 10 (*n* = 9 after removing significant outlier); APO-SUS + NAC, *n* = 10; APO-UNSUS, *n* = 9; APO-UNSUS + NAC, *n* = 10) and qPCR analyses (see Supplementary Tables 3 and 4 for *n*-numbers).

### Immunohistochemistry

For immunohistochemistry, APO-SUS and APO-UNSUS rats of P90 (±1 day) with and without NAC treatment were perfused with 2% paraformaldehyde (PFA; oligodendrocyte (OL) lineage cell number APO-SUS, *n* = 5 and *n* = 4 after removing a significant outlier; APO-SUS + NAC, *n* = 6; APO-UNSUS, *n* = 7; APO-UNSUS + NAC, *n* = 10; OL precursor cell (OPC) number APO-SUS, *n* = 4; APO-SUS + NAC, *n* = 7; APO-UNSUS, *n* = 5; APO-UNSUS + NAC, *n* = 7; mature OL number APO-SUS, *n* = 6 and *n* = 5 after removing a significant outlier; APO-SUS + NAC, *n* = 7; APO-UNSUS, *n* = 10; APO-UNSUS + NAC, *n* = 4). Perfused brains were removed, postfixed overnight, and placed in 30% sucrose in PBS for 3–5 days, frozen on dry ice and stored at −80 °C until further processing. Coronal cryosections of 10 μm were collected in a cryostat (Leica) and rehydrated in 1× PBS. Antigen retrieval was performed in a microwave using citric acid-based antigen unmasking solution (Vector) for OLIG2-CC1 immunostaining. Tissue was blocked in 4% BSA, 0.1% Triton X-100 for 1 h at room temperature (RT). Primary antibodies were anti-OLIG2 (AB9610, Millipore 1:1000 or  MABN50, Millipore 1:1000), anti NG2 (AB5320, Millipore 1:100) and anti-CC1 (OP80, Calbiochem 1:100) and incubated overnight at 4 °C. Secondary antibodies were 488, 555 and 568-conjugated anti-rabbit or anti-mouse (Alexa 1:1000)  and incubated for 2 h at RT. Hoechst (H6024, Sigma 1:1000) was added as a nuclear counterstain. Sections were mounted in Fluoromount (0100-01, Southern Biotech) and ×20 images were obtained using an Axioscan (Leica) and analysed with Zen (Blue edition). Regions of interest were drawn and cells were counted manually.

### Primary oligodendroglial cell cultures

Mixed glia cultures were obtained from cortex of P1 APO-SUS and APO-UNSUS rats, *n* = 3 replicates per condition. The tissue was homogenized in mixed glia culture medium (GlutaMAX (Invitrogen), 10% fetal bovine serum (Thermo Fisher) 1% Pen-Strep (Thermo Fisher) and 1% nonessential amino acids (Thermo Fisher)) and cells were kept in this medium on 1:10 poly-L ornithine (PLO) coated T75 flasks at 37 °C 5% CO_2_. Medium was refreshed after 7 and 13 days in culture and at day 14 OPCs were purified using a shaking protocol. Mixed glia cultures were shook at 250 rpm for 1 h to discard microglia cells, then cultures were shook again at 250 rpm for 18 h to purify OPCs. Supernatant containing OPCs was placed on petridishes (Falcon) three times for 5 min to eliminate astrocyte contamination. OPCs were then plated onto PLO-coated coverslips in 24-well plates. After 4 h, medium was changed to differentiation medium for 4 days (DMEM-F12 (Invitrogen), 0.5% B27 (Sigma) 1% Pen-Strep (Thermo Fisher) and 0.05% 40 ng/mL of T3 thyroid hormone (Sigma)) with or without 1 µM cobalt chloride hexahydrate (CoCl_2_ 6(H_2_O)), a chemical that causes oxidative stress. After 4 days, cultures were stained with homemade O4 antibody for 1 h and fixed in 2% PFA for 7 min. Subsequently OPCs were incubated with anti-SOX10 (1/100, R&D Systems AF2864) and anti-MBP (1/200, Abcam ab7349) primary antibodies for 1 h at RT, washed with 1× PBS and incubated with secondary antibodies (donkey anti-TRITC IgM 1/100 (Southern Biotech), donkey anti-goat Alexa 488 1/1000, donkey anti-rat Alexa 647 1/750 and Hoechst) for 1 h at RT, washed and mounted with fluoromount. Images were obtained in an Axioscan and analysed in Zen (Bleu edition) manually.

### Operant attentional set-shifting tests

Upon the start of behavioral training, rats were food restricted and received 5–7 g of food per 100 g of rat daily (APO-SUS, *n* = 29; APO-UNSUS, *n* = 28; APO-SUS + NAC, *n* = 10; APO-UNSUS + NAC, *n* = 10; the number of untreated APO-SUS and APO-UNSUS control rats was higher than the number of NAC-treated rats since we included untreated APO-SUS and APO-UNSUS control animals from other cognitive-flexibility-related behavioral experiments performed during the same time period). Rats were pre-exposed once to grain reward pellets (Rodent Tablet [5TUM], 45 mg, TestDiet, USA) in the home cage. Operant conditioning chambers (29.5 cm l, 24 cmW, 25 cm H; Med Associates, Georgia, VT) were situated in light and sound-attenuating cubicles equipped with a ventilation fan. Each chamber was equipped with two 4.8-cm-wide retractable levers, placed 11.7 cm apart and 6 cm from the grid floor. A cue light (28 V, 100 mA) was present above each lever. At the same wall, a reward pellet could be delivered in a magazine between the levers and a house light (28 V,100 mA) was located on the same wall. The lever presentation and cue light illumination sides were counterbalanced between rats. Pretraining, operant extra-dimensional set-shifting and reversal learning were performed as described previously [16]. Rats were trained to acquire the initial set (lever below the cue light is correct) and subsequently performed three extra-dimensional set-shifts (note that between extra-dimensional shifts 2 and 3 reversal learning was performed). As we wanted to examine the rats’ ability to shift a set repeatedly, rats reacquired the initial set before each set-shift session and the first 20 trials of the set-shift sessions were following the initial set, while from trial 21 onwards rats were required to make the extra-dimensional shift and press the lever on one side of the cage regardless of cue light illumination. During reversal learning rats were required to switch from pressing the lever on one side of the cage to pressing the lever on the other sise of the cage regardless of cuelight illumination. We measured the number of errors made until a criterion of ten subsequent correct trials was reached and classified the errors as perseverative errors (following the ‘old rule’), regressive errors (following the ‘old rule’ while more than 70% of previous trials were correct) or never-reinforced errors (pressing a lever that was incorrect during both the ‘old rule’ and during the current rule).

### Statistical analyses

For qPCR, glutathione assay and electron microscopy analysis, statistical significance was calculated using the Independent samples *T*-test and, where appropriate, with Benjamini–Hochberg correction for multiple comparisons. Linear regression with Two-way ANOVA was used to test statistical significance of the regression between G-ratio and axon caliber. The mRNA expression data of the NAC-treatment experiment was analysed using Two-way multivariate ANOVA with rat line and treatment as independent variables and the mRNA expression values of the genes examined as dependent variables. We did multivariate ANOVAs rather than univariate ANOVAs on the mRNA expression data because the genes examined are involved in the same biological network (see Fig. 2d) and therefore not completely independent of each other. Immunohistochemistry, electron microscopy, glutathione assay, and attentional set-shift data analyses of the NAC-treatment and CoCl_2_-treatment experiments were performed using Two-way ANOVA *F*-tests based on type II sum of squares. Significance for the main effects of rat line (APO-SUS versus APO-UNSUS), treatment (NAC versus no NAC or CoCl_2_ versus no CoCl_2_) and their interaction were evaluated using *F*-tests and in case of a significant interaction effect post-hoc pairwise comparisons were evaluated using Tukey’s test. Two-way ANOVA data analyses were performed in R version 3.6.1 (R Development Core Team, 2019) using the ANOVA function in the car package and Tukey’s method in the Emmeans package. All other statistical analyses were performed using IBM SPSS Statistics 24. For all analyses, outliers were discarded beforehand as indicated by the Grubbs outlier test using Graphpad quickcalcs, and the level of significance was set at *p* = 0.05.

## Results

### Oxidative stress in APO-SUS mPFC

**Fig. 1:**
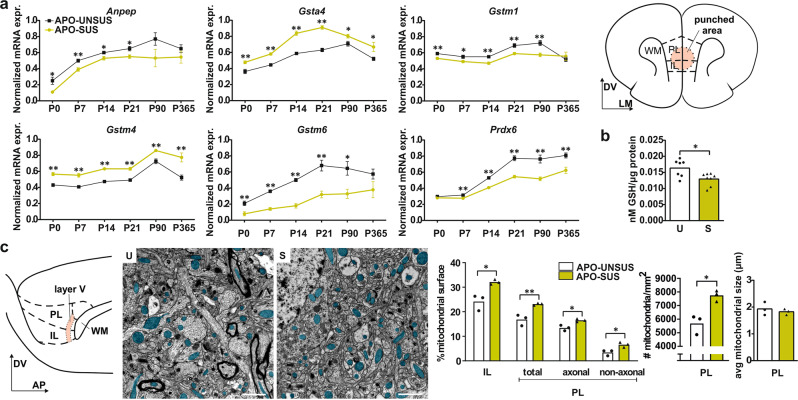
Oxidative stress in APO-SUS versus APO-UNSUS mPFC. **a** mRNA expression of alanyl aminopeptidase-N (*Anpep*), glutathione-s-transferase-α4 (*Gsta4*), glutathione-s-transferase-µ1 (*Gstm1*), glutathione-s-transferase-µ4 (*Gstm4*), glutathione-s-transferase-µ6 (*Gstm6*), and peroxiredoxin 6 (*Prdx6*) in APO-SUS (S) and APO-UNSUS (U) mPFC. See Supplementary Table 2 for exact *p* values. Schematic representation of coronal mPFC section adapted from the Paxinos and Watson rat brain atlas in which the area included in the micropunch used for qPCR and GSH analyses is indicated. DV dorso-ventral axis, LM lateral-medial axis. Micro punched tissue was used for data in (**a**, **b**). **b** Glutathione (GSH) levels (nMol per µg protein) in APO-SUS versus APO-UNSUS mPFC. **c** Schematic representation of sagittal mPFC section adapted from the Paxinos and Watson rat brain atlas indicating mPFC layer 5 in IL and PL subregions. DV dorso-ventral axis, AP anterior-posterior axis. Representative images and analyses of APO-SUS and APO-UNSUS IL, PL, PL axonal and PL non-axonal mitochondrial surface areas, exact mitochondria count and mitochondrial size. Blue overlay indicates mitochondria. Scale bars 1 µm; *p < 0.05 and **p < 0.01 in independent samples *T*-test with Benjamini–Hochberg multiple comparisons correction. Error bars represent standard error of the mean.

RNA-seq analysis of APO-SUS and APO-UNSUS mPFC revealed 858 differentially expressed genes (DEGs; fold change ≥1.2, likelihood ratio test corrected *p* < 0.05; 371 genes upregulated and 487 genes downregulated; see Supplementary Dataset). IPA revealed that the two top-enriched canonical pathways were ‘glutathione-mediated detoxification’ and ‘glutathione redox reactions I’ (*p* = 9.24E−10 and ratio = 0.258, and *p* = 1.52E−04 and ratio = 0.25, respectively, in Benjamini–Hochberg corrected *T*-test; Supplementary Tables 5-7). qPCR analysis confirmed a dysregulation of glutathione-related genes from these two pathways in the mPFC of APO-SUS rats that started early in development (P0) and persisted into late adulthood (P365) (Fig. 1a). Furthermore, a kinetic assay showed significantly lower levels of glutathione in APO-SUS versus APO-UNSUS mPFC (Fig. 1b; Independent samples *T*-test *t*[13] = −2.817, *p* = 0.015). Thus, glutathione antioxidant metabolism was reduced in APO-SUS mPFC throughout postnatal development and adulthood. In addition, the top 35 DEGs in APO-SUS mPFC encompassed five downregulated myelin-related mRNAs (Supplementary Table 8), in line with mPFC hypomyelination in APO-SUS rats [16]; note that myelin-related genes do not constitute a canonical pathway in the IPA software package. On a further note, the degree of the differential expression of the genes comprising the glutathione metabolism pathway is mostly lower than that of the top 35 genes, but this set of genes was identified by IPA since it represents a significant phenomenon of biological importance.

Mitochondria play a key role in the formation of oxidative stress and in SZ oxidative stress is associated with alterations in mitochondria [21]. Electron microscopy revealed an increased surface area containing mitochondria in APO-SUS as compared to APO-UNSUS IL and PL mPFC subregions (Fig. 1c; Independent samples *T*-test IL *t*[4] = 4.056, *p* = 0.015, PL *t*[4] = 4.809, *p* = 0.009). As the difference was largest in the PL subregion, further analyses were performed only in this mPFC subregion. APO-SUS rats have an increased surface area containing mitochondria in both axonal and non-axonal structures, indicating that the increase in mitochondrial surface in APO-SUS mPFC was not specific to neuronal axons (Fig. 1c; Independent samples *T*-test axonal mitochondrial surface: *t*[4] = 4.235, *p* = 0.013, non-axonal mitochondrial surface: *t*[4] = 3.543, *p* = 0.024). Mitochondrial sizes were not different (Fig. 1c; Independent samples *T*-test *t*[4] = −0.687, *p* = 0.5304), but rather the number of mitochondria was increased in APO-SUS mPFC (Fig. 1c; Independent samples *T*-test *t*[4] = 4.340, *p* = 0.012).

### NAC treatment alleviates oxidative stress in APO-SUS mPFC

**Fig. 2:**
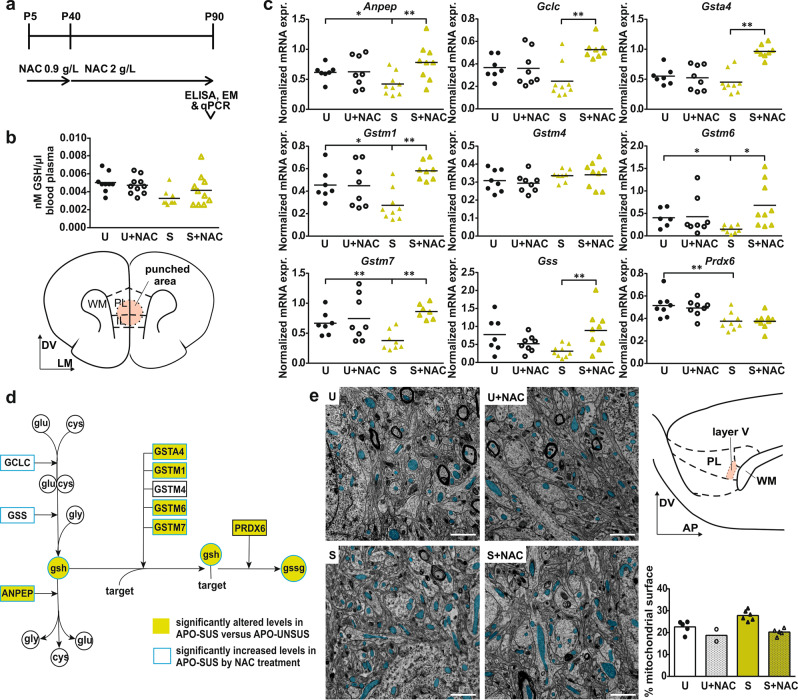
NAC treatment alleviates oxidative stress in APO-SUS mPFC. **a** Schematic representation of experimental paradigm. **b** Glutathione (GSH) levels (nanoMol) per microliter blood plasma in APO-SUS (S) and APO-UNSUS (U) rats with and without NAC treatment. **c** mRNA expression of *Anpep*, glutathione cysteine ligase catalytic subunit (*Gclc*), glutathione synthetase (*Gss*), *Gsta4, Gstm1*, *Gstm4*, *Gstm6*, *Gstm7*, and *Prdx6* in mPFC of APO-SUS and APO-UNSUS rats with and without NAC treatment. For statistical values, see Supplementary Table 3. Schematic representation of coronal mPFC section adapted from the Paxinos and Watson rat brain atlas in which the area included in the micropunch used for qPCR analyses is indicated. DV dorso-ventral axis, LM lateral-medial axis. **d** Schematic representation of GSH metabolic pathway. White circles = amino acids, coloured circles = tripeptides, squares = enzymes, green symbols refer to components with altered levels in APO-SUS versus APO-UNSUS mPFC, blue outlines refer to components with significantly increased levels in APO-SUS after NAC treatment. **e** Representative images and analysis of mitochondrial surface in mPFC of APO-SUS and APO-UNSUS rats with and without NAC treatment. Blue overlay indicates mitochondria. Schematic representation of sagittal mPFC section adapted from the Paxinos and Watson rat brain atlas with PL layer V indicated where EM analyses were performed. DV dorso-ventral axis, AP anterior-posterior axis. Scale bars 1 µm; *p < 0.05, **p < 0.01 in Independent samples *T*-test with Benjamini–Hochberg multiple comparisons correction.

We next wondered whether treatment with the glutathione precursor NAC from P5 onwards could restore the APO-SUS mPFC alterations in glutathione metabolism and mitochondria numbers (Fig. 2a). NAC treatment did not significantly affect glutathione levels in the blood of APO-SUS or APO-UNSUS rats (Fig. 2b; Two-way ANOVA interaction *F*[1,37] = 2.264, *p* = 0.142, main effects rat line *F*[1,37] = 8.072, *p* = 0.007 treatment *F*[1,37] = 0.571, *p* = 0.455). However, in the mPFC NAC treatment significantly changed APO-SUS mRNA expression levels of a number of glutathione-related genes to APO-UNSUS levels (Fig. 2c; Two-way multivariate ANOVA interaction Wilks’ Lambda *F*[9] = 5.214, *p* = 0.003, main effects rat line Wilks’ Lambda *F*[9] = 16.236, *p* < 0.001 treatment Wilks’ Lambda *F*[9] = 3.839, *p* = 0.011), including genes involved in the production and breakdown of glutathione, as well as the binding of glutathione to target oxidative molecules (Fig. 2d). In APO-UNSUS rats, NAC treatment did not affect mPFC glutathione-related mRNA expression (Fig. 2c). Moreover, ultrastructural analysis showed that the mitochondrial surface in the mPFC of NAC-treated APO-SUS and APO-UNSUS rats was decreased (Fig. 2e; Two-way ANOVA interaction *F*[1,17] = 2.033, *p* = 0.176, main effects rat line *F*[1,17] = 10.26, *p* = 0.006 treatment *F*[1,17] = 26.43, *p* < 0.001). We conclude that NAC treatment during postnatal development alleviates oxidative stress in the APO-SUS mPFC.

### NAC treatment improves hypomyelination in APO-SUS mPFC

**Fig. 3:**
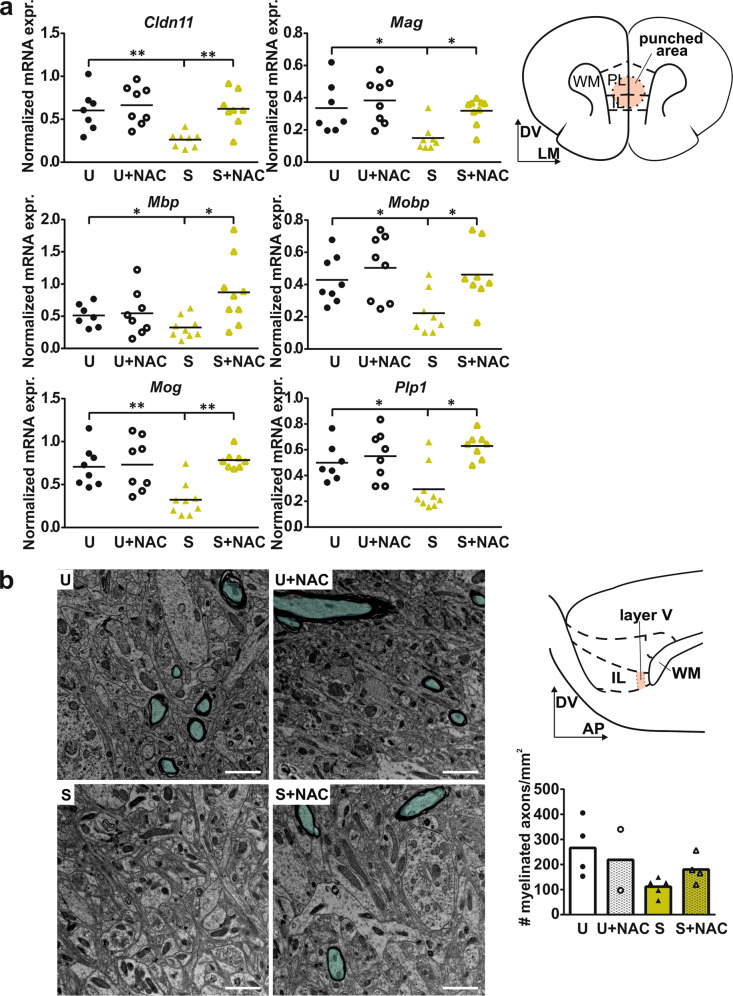
NAC treatment improves hypomyelination in APO-SUS mPFC. **a** Normalized mRNA expression of claudin 11 (*Cldn11*), myelin associated glycoprotein (*Mag*), myelin basic protein (*Mbp*), myelin oligodendrocyte basic protein (*Mobp*), oligodendrocyte glycoprotein (*Mog*), proteolipid protein 1 (*Plp1*) in mPFC of APO-SUS (S) and APO-UNSUS (U) rats with and without NAC treatment. For exact statistical values see Supplementary Table 4. Schematic representation of coronal mPFC section adapted from the Paxinos and Watson rat brain atlas in which the area included in the micropunch used for qPCR analyses is indicated. DV dorso-ventral axis, LM lateral-medial axis. **b** Representative images and quantification of the number of myelinated axons in APO-SUS and APO-UNSUS rats with and without NAC treatment. Turquoise overlay indicates myelinated axons. Scale bars 1 µm. Schematic representation of saggital mPFC section adapted from the Paxinos and Watson rat brain atlas indicating IL layer V that was used for EM analysis. DV dorso-ventral axis, AP anterior-posterior axis. *p < 0.05 **p < 0.01 in Independent samples *T*-test with Benjamini–Hochberg multiple comparisons correction.

Hypomyelination of mPFC interneurons occurs in APO-SUS rats during adolescence [16]. Oxidative stress is thought to affect the maturation of PFC interneurons in adolescent SZ patients [22]. However, it remains unclear whether redox imbalance is a causative factor for interneuron hypomyelination. Strikingly, NAC treatment during postnatal development normalized the mRNA expression of all major myelin components in APO-SUS mPFC (Fig. 3a; Two-way multivariate ANOVA interaction Wilks’ Lambda *F*[6] = 3.050, *p* = 0.031, main effects rat line Wilks’ Lambda *F*[6] = 1.813, *p* = 0.153 treatment Wilks’ Lambda *F*[6] = 2.553, *p* = 0.057). Our ultrastructural analysis did not reveal a significant effect of NAC treatment on the number of myelinated axons in APO-SUS IL (Fig. 3b; Two-way ANOVA interaction *F*[1,14] = 1.466, *p* = 0.251, main effects rat line *F*[1,14] = 5.714, *p* = 0.036, treatment *F*[1,14] = 0.294, *p* = 0.598), likely due to the low sample sizes commonly used in electron microscopy experiments. Myelin thickness as measured by the G-ratio remained unaffected (Supplementary Fig. S1). Therefore, we conclude that NAC treatment improves the hypomyelination phenotype in the mPFC of APO-SUS rats without affecting myelin structure.

### Oxidative stress hinders APO-SUS OL maturation

**Fig. 4:**
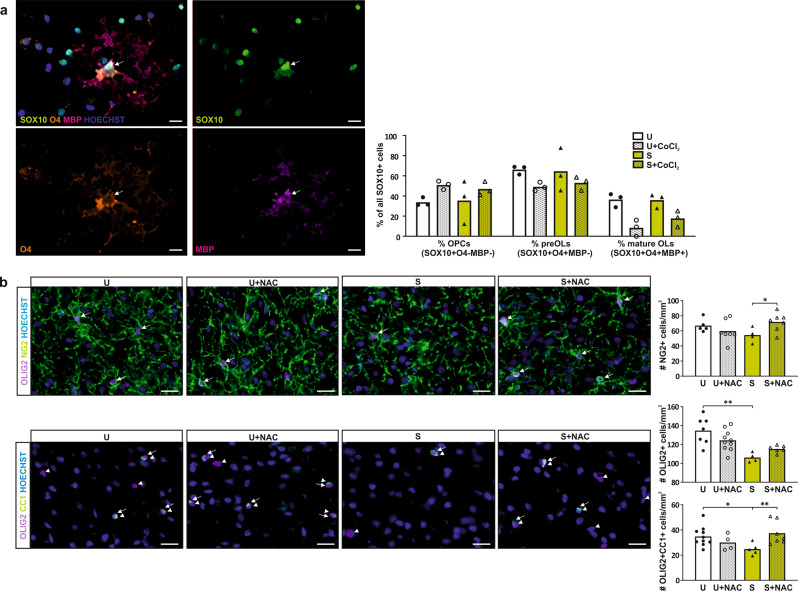
NAC treatment rescues OL lineage progression in APO-SUS mPFC. **a** Representative image and quantification of the percentages of all SOX10 + oligodendroglia cells that are OPC (SOX10 + O4-MBP−), premyelinating OL (preOL) (SOX10 + O4 + MBP−) and mature OL (SOX10 + O4 + MBP + ) in APO-SUS (S) versus APO-UNSUS (U) primary oligodendroglia cultures treated with and without CoCl_2_ 6(H_2_O) (CoCl_2_). Arrows indicate SOX10 + O4 + MBP + mature OL. **b** Representative images and quantification of the number of OPCs (NG2+ indicated by arrows), OL lineage cells (OLIG2+ indicated arrowheads in OLIG2CC1 images) and mature OLs (OLIG2 + CC1 + indicated by arrows) per mm^2^ in the IL of APO-SUS versus APO-UNSUS rats with and without NAC treatment. Scale bars 20 µm; *p < 0.05 **p < 0.01 in Two-way ANOVA post-hoc pairwise comparisons using Tukey’s test.

Interneuron hypomyelination in APO-SUS mPFC is caused by impaired OL lineage progression [16]. OLs and in particular premyelinating OLs are highly sensitive to oxidative stress [23–25]. As glutathione metabolism is altered from P0 onwards and myelination of mPFC interneurons takes place during adolescence, we hypothesized that prolonged oxidative insult to OLs prior to and during adolescence, hinders proper myelination of the mPFC. To test this hypothesis, we differentiated primary OPCs, isolated from newborn APO-SUS and APO-UNSUS rat cortices, in the presence or absence of 1 µM CoCl_2_, a chemical that causes oxidative stress, and analyzed OL lineage progression. The intrinsic capacity of APO-SUS and APO-UNSUS OPCs to mature in vitro was not different (Fig. 4a). However, CoCl_2_ significantly reduced the percentage of mature OLs in differentiated APO-SUS and APO-UNSUS OPC cultures (Fig. 4a; Two-way ANOVA interaction *F*[1,11] = 1.323, *p* = 0.283, df = 1, main effects rat line *F*[1,11] = 1.043, *p* = 0.337 treatment *F*[1,11] = 28.941, *p* < 0.001). In addition, there was a trend towards an increased percentage of OPCs, and a decreased percentage of preOLs in APO-SUS and APO-UNSUS OPC cultures exposed to CoCl_2_ (Fig. 4a; preOLs Two-way ANOVA interaction *F*[1,11] = 0.176, *p* = 0.686, main effects rat line *F*[1,11] = 0.026, *p* = 0.875 treatment *F*[1,11] = 4.527, *p* = 0.066, OPCs Two-way ANOVA interaction effect *F*[1,11] = 0.176, *p* = 0.686, main effects rat line *F*[1,11] = 0.026, *p* = 0.875 treatment *F*[1,11] = 4.527, *p* = 0.066). We therefore conclude that oxidative stress hinders the maturation of APO-SUS and APO-UNSUS OPCs in vitro. In line with these in vitro data, we found in vivo a reduced number of OLIG2 + OL lineage cells and mature OLIG2 + CC1 + OLs in the mPFC of oxidative-stress-exposed APO-SUS rats as compared to the mPFC of APO-UNSUS rats. Following NAC treatment, the numbers of OPCs and mature OLs in the APO-SUS mPFC were significantly increased (Fig. 4b; OL lineage cells Two-way ANOVA interaction *F*[1,26] = 5.228, *p* = 0.032 main effects rat line *F*[1,26] = 16.68, *p* < 0.001 treatment *F*[1,26] = 0.602, *p* = 0.446, post-hoc pairwise comparisons APO-SUS versus APO-UNSUS *t*[23] = −4.725, *p* < 0.001; OPCs Two-way ANOVA interaction effect *F*[1,22] = 5.806, *p* = 0.026 main effects rat line *F*[1,22] = 0.252, *p* = 0.621 treatment *F*[1,22] = 0.695, *p* = 0.415; post-hoc pairwise comparisons APO-SUS versus APO-SUS + NAC *t*[9] = −2.33, *p* = 0.031; mature OLs Two-way ANOVA interaction *F*[1,25] = 7.680, *p* = 0.011 main effects rat line *F*[1,25] = 0.649, *p* = 0.429 treatment *F*[1,25] = 1.614, *p* = 0.217, post-hoc pairwise comparisons APO-SUS versus APO-UNSUS *t*[22] = 2.43, *p* = 0.0237 APO-SUS versus APO-SUS + NAC *t*[22] = −2.85, *p* = 0.009), indicating that the promyelinating effects of the antioxidant are, at least partially, caused by a positive effect on OL lineage progression.

### NAC treatment improves mPFC-dependent cognitive inflexibility in APO-SUS rats during extra-dimensional set-shifting and reversal learning

**Fig. 5:**
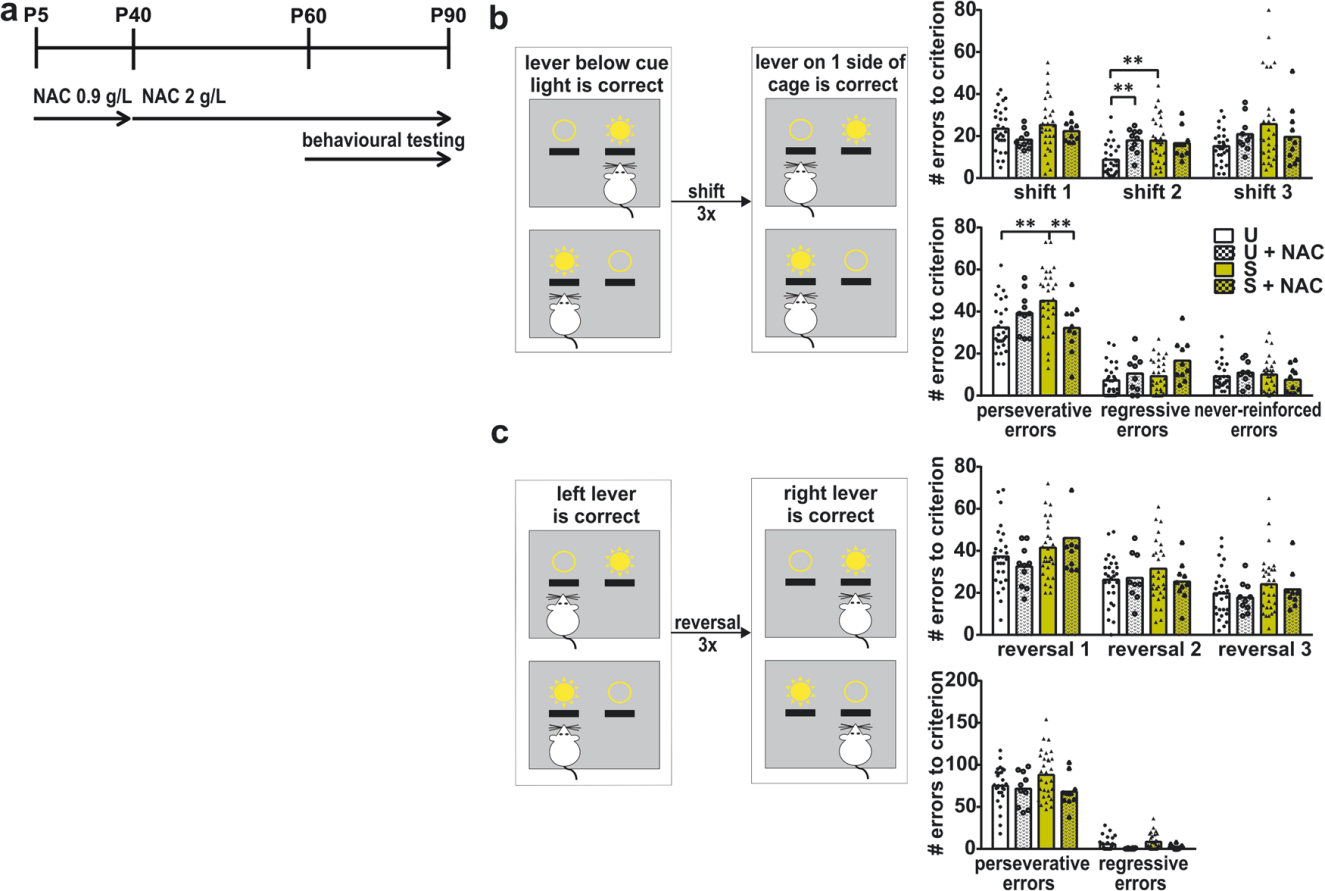
NAC treatment improves cognitive inflexibility in APO-SUS rats during extra-d**i**mensional set-shifting and reversal learning. **a** Schematic representation of the experimental design and **b** extra-dimensional set-shifting task. Rats were trained to press the lever above which a cue light was illuminated. From trail 21 of the next session onwards, rats were required to press the lever on one side of the cage irrespective of the cue light. Number of errors to criterion in set-shift 1, 2, and 3, as well as the total number of perseverative, regressive, and never-reinforced errors over the three shifts in APO-UNSUS (U) and APO-SUS (S) rats with and without NAC treatment are depicted. **c** Schematic representation of reversal learning. Rats were required to press the lever on one side of the cage regardless of cue light illumination. During the reversal sessions, rats had to press the lever on the other side of the cage. Number of errors to criterion in reversals 1, 2, and 3, as well as the total number of perseverative, regressive, and never-reinforced errors over the three reversals in APO-UNSUS and APO-SUS rats with and without NAC treatment are depicted. *p < 0.05 **p < 0.01 in Two-way ANOVA post-hoc pairwise comparisons using Tukey’s test.

We next wondered whether alleviating oxidative stress and restoring myelination in the APO-SUS mPFC would lead to improved cognitive behavior. We therefore treated APO-SUS and APO-UNSUS rats with and without NAC, and performed the extra-dimensional set-shifting task that is modeled after the Wisconsin card sorting test used to examine cognitive symptoms in SZ patients [26, 27]. During initial training to press the levers as well as during training to press the lever below the illuminated cue light (the acquisition of the initial rule), APO-SUS and APO-UNSUS rats performed similarly (Supplementary Fig. S2a, b). During the extra-dimensional set-shifting sessions, we found that all rats performed well above chance level during the first 20 trials of each session (which comprised the initial rule), although APO-SUS rats had a higher percentage of correct trials than APO-UNSUS rats during the first 20 trials of shift 2 (Supplementary Fig. S2c). After trial 21, rats had to make the extra-dimensional shift by pressing the lever on one side of the cage to get a reward (shifting to a “new rule”) and we found that during shifts 2 and 3 APO-UNSUS rats made significantly less errors to criterion (streak of 10 correct trials) than APO-SUS rats. During shift 1 NAC treatment decreased the number of errors to criterion made by both APO-SUS and APO-UNSUS rats and during shift 2 NAC treatment increased the number of errors to criterion made by APO-UNSUS rats (Fig. 5a, b, see also [16]; Shift 1 Two-way ANOVA interaction *F*[1,77] = 0.003, *p* = 0.956 main effects rat line *F*[1,77] = 0.221, *p* = 0.640 treatment *F*[1,77] = 0.835, *p* = 0.044; Shift 2 Two-way ANOVA interaction *F*[1,77] = 5.774, *p* = 0.019 main effects rat line *F*[1,77] = 11.69, *p* = 0.001 treatment *F*[1,77] = 3.39, *p* = 0.070, post-hoc pairwise comparisons APO-SUS versus APO-UNSUS *t*[74] = −4.17, *p* < 0.001 APO-UNSUS versus APO-UNSUS + NAC *t*[74] = −3.003, *p* = 0.003; Shift 3 Two-way ANOVA interaction *F*[1,77] = 2.129, *p* = 0.149 main effects rat line *F*[1,77] = 4.479, *p* = 0.038 treatment *F*[1,77] = 0.011, *p* = 0.917). However, error analysis revealed that untreated APO-SUS rats made significantly more perseverative errors than untreated APO-UNSUS rats during all three shifts combined, indicating that APO-SUS rats kept following the initial rule and showed cognitive inflexibility. NAC treatment rescued the number of perseverative errors in APO-SUS rats, while not affecting perseverative error numbers of APO-UNSUS rats (Fig. 5a, b; Perseverative errors Two-way ANOVA interaction *F*[1,77] = 8.493, *p* = 0.005 main effects rat line *F*[1,77] = 6.574, *p* = 0.012 treatment *F*[1,77] = 0.836, *p* = 0.363, post-hoc pairwise comparisons APO-SUS versus APO-UNSUS *t*[74] = −3.69, *p* < 0.001 APO-SUS versus APO-SUS + NAC *t*[74] = 2.701, *p* = 0.008; Regressive errors Two-way ANOVA interaction *F*[1,77] = 1.071, *p* = 0.304 main effects rat line *F*[77] = 2.365, *p* = 0.128 treatment *F*[77] = 6.744, *p* = 0.011; Never-reinforced errors Two-way ANOVA interaction effect *F*[77] = 1.361, *p* = 0.247 main effects rat line *F*[77] = 0.023, *p* = 0.879 treatment *F*[77] = 0.041, *p* = 0.840). NAC treatment also increased the number of regressive errors in APO-SUS and APO-UNSUS rats, but these rats make five times more perseverative errors than regressive errors and as such the effect of NAC treatment is twice as large on perseverative errors than on regressive errors.

To further investigate the effects of NAC on cognitive inflexibility, we performed reversal learning. Rats were trained to press the lever on one side of the cage regardless of cue light illumination and during the reversal sessions, rats were required to reverse their behavior and press the lever on the other side of the cage. We quantified the number of errors the rats made until they reached the criterion in three subsequent reversal sessions (streak of 10 correct trials). We found a trend towards an increased number of errors to criterion in APO-SUS rats during reversal 1, but no further differences in the performance of APO-SUS and APO-UNSUS rats during reversals 2 and 3 (Fig. 5c; Reversal 1 Two-way ANOVA interaction *F*[1,77] = 1.394, *p* = 0.242 main effects rat line *F*[1,77] = 3.634, *p* = 0.061 treatment *F*[1,77] = 0.001, *p* = 0.983; Reversal 2 Two-way ANOVA interaction *F*[1,76] = 0.874, *p* = 0.353 main effects rat line *F*[1,76] = 1.213, *p* = 0.274 treatment *F*[1,76] = 0.562, *p* = 0.456; Reversal 3 Two-way ANOVA interaction *F*[1,76] = 0.004, *p* = 0.948 main effects rat line *F*[1,76] = 2.392, *p* = 0.126 treatment *F*[1,76] = 0.487, *p* = 0.488). However, during error analysis we found that NAC treatment causes a trend towards a lower number of perseverative errors as well as a significant decrease in the number of regressive errors in APO-SUS and APO-UNSUS rats (Fig. 5c; Perseverative errors Two-way ANOVA interaction *F*[1,76] = 1.737, *p* = 0.192 main effects rat line *F*[1,76] = 2.523, *p* = 0.117 treatment *F*[1,76] = 3.565, *p* = 0.063; Regressive errors Two-way ANOVA interaction effect *F*[1,76] = 0.017, *p* = 0.898 main effects rat line *F*[1,76] = 2.441, *p* = 0.123 treatment *F*[1,76] = 8.209, *p* = 0.005). Thus, NAC treatment improved cognitive flexibility in APO-SUS rats in both extra-dimensional set-shifting and reversal learning cognitive tasks.

## Discussion

Animal models recapitulating (some of) the positive, negative, and cognitive symptoms of SZ are instrumental to understand the molecular and cellular mechanisms underlying these symptoms. In this study, we used the APO-SUS rat model displaying SZ-relevant cognitive inflexibility to explore the elusive neurobiological basis of PFC-related cognitive symptoms in SZ. Unbiased transcriptomic analysis revealed that genes related to glutathione metabolism were enriched among the genes differentially expressed in mPFC of APO-SUS versus APO-UNSUS rats. The observed reduced levels of glutathione in APO-SUS mPFC were accompanied by an increase in the number of mitochondria. Oxidative stress and mitochondrial dysfunction have been hypothesized to play a role in SZ etiology [28]. In individuals at high risk to develop SZ, mitochondrial dysfunction has been linked to symptoms of the disorder [29], indicating that mitochondrial abnormalities are present already before disease onset. As genetic risk for SZ includes genetic variations in both mitochondria- and oxidative stress-related genes [21], it is unclear whether mitochondrial dysfunction arises as a consequence of oxidative stress or induces oxidative stress. The fact that in APO-SUS rats we found impaired glutathione metabolism already at birth that persisted into late adulthood suggesting that oxidative stress in SZ may occur before the clinical symptoms of the disorder become apparent.

Reduced PFC glutathione levels have been associated with decreased PFC white-matter integrity that is thought to arise from defective myelination [5]. Expression of myelin-related mRNAs and proteins is decreased in postmortem SZ frontal cortex [30, 31] and myelin content is reduced in frontal areas of SZ patients [32]. In APO-SUS rats, hypomyelination occurs during adolescent mPFC development and predominantly in interneurons [16]. Notably, during adolescent mPFC development interneurons undergo maturational changes that include myelination and the formation of perineuronal nets, both of which are thought to be affected in SZ by oxidative stress [22].

To investigate whether oxidative stress causes hypomyelination in the APO-SUS mPFC, we chronically treated postnatal APO-SUS rats with the antioxidant glutathione precursor NAC. We found normalized mPFC glutathione metabolism-related mRNA expression levels in adult NAC-treated APO-SUS rats as well as a reduced number of mPFC mitochondria in NAC-treated APO-SUS and APO-UNSUS rats. Remarkably, besides its antioxidative properties, NAC also showed promyelinating effects in that the mRNA expression of all major myelin components was significantly increased in the mPFC of APO-SUS rats treated with the antioxidant. Thus, a proper glutathione metabolism appears to be essential for developmental myelination in SZ. The notion that oxidative stress underlies PFC hypomyelination in SZ is corroborated by the results of our analysis of the developmental time course of mPFC mRNA expression, which showed that the dysregulation of glutathione-related genes preceded the reduced expression of myelin-related genes in APO-SUS rats.

Since OLs, and in particular premyelinating OLs, are extremely vulnerable to oxidative stress [24, 25], we hypothesized that interneuron hypomyelination in SZ may be caused by adverse effects of oxidative stress on OL lineage progression. OL maturation is impaired in APO-SUS mPFC [16] and we here find that NAC increases the number of OPCs and mature OLs in APO-SUS mPFC. Moreover, our in vitro experiments with APO-SUS and APO-UNSUS OPCs showed that an oxidative insult is indeed capable of impairing OPC maturation. The fact that NAC treatment positively influenced OL lineage progression supports the notion that oxidative damage to OLs contributes to hypomyelination in the APO-SUS mPFC and may well contribute to hypomyelination in SZ.

APO-SUS rats show mPFC-dependent cognitive inflexibility in the extra-dimensional set-shifting task [16]. This cognitive inflexibility was characterized by a perseveration of previously learned behavior. In the current study, we found only a trend towards impaired performance during reversal 1, but no further evidence for a reversal learning impairment in APO-SUS rats, while we previously found an increased perseveration in this test [16]. In SZ patients, both set-shifting and reversal learning impairments are evident and characterized by an increased perseveration [27, 33, 34]. The operant paradigm we used to test cognitive inflexibility is a rodent version of the Wisconsin card sorting test that is frequently used to reveal extra-dimensional set-shifting impairments in SZ patients and in SZ animal models [35, 36]. For example, in the neonatal ventral hippocampal lesioned animal model of SZ the identified attentional set-shifting impairments were also characterized by increased perseveration [37]. In addition, the PCP rat model of SZ [38] as well as a mouse SZ model with NPAS4 deficiency and chronic mild stress [39] showed attentional set-shifting defects. Intriguingly, the antioxidative and promyelinating effects of NAC in APO-SUS mPFC led to a significant improvement in cognitive inflexibility during extra-dimensional set-shifting. NAC treatment also revealed a trend towards less perseveration in both APO-SUS and APO-UNSUS rats during reversal learning. As an add-on treatment to antipsychotic medication, NAC has moderate effects on the positive, negative, and cognitive symptoms of chronic SZ patients [40–42]. The moderate effects of NAC on cognition were replicated in early-psychosis patients and accompanied by increases in PFC glutathione levels [43].

Collectively, the findings increase our understanding of the neurobiological mechanisms leading to cognitive symptoms in SZ and encourage the use of chronic NAC treatment as a preventive measure for individuals at high risk for developing SZ and early-phase SZ patients.

## Funding and disclosure

The authors declare that they have no conflict of interest. This study was supported by grants from the programs ‘Top talent’ Donders Centre for Medical Neuroscience (to DAM), Van Gogh travel grant (to DAM, BNO and GJMM), Investissements d’Avenir ANR-10-IAIHU-06 (IHU-A-ICM) and ANR-11-INBS-0011 (NeurATRIS) (to BNO) and Dutch Top Institute Pharma T5-209 (to VDE and GJMM).

## Supplementary information

Supplemental Material

Supplementary Dataset
