# Supplementary material for: Antioxidant treatment ameliorates prefrontal hypomyelination and cognitive deficits in a rat model of schizophrenia
Source: Neuropsychopharmacology. 2021 Feb 9;46(6):1161–71. doi: 10.1038/s41386-021-00964-0 (PMC8115238; doi:10.1038/s41386-021-00964-0)
Supplement: Supplementary file 1 — Supplemental Material [file 41386_2021_964_MOESM1_ESM.pdf]

Supplementary Material

Maas et al., Antioxidant treatment ameliorates prefrontal hypomyelination and cognitive deficits in a rat model of schizophrenia

Supplementary Table 1 – Primer sequences.

Gene and abbreviation		Forward primer 5'-3'	Reverse primer 5'-3'
**Housekeeping genes**			
β-actin	Beta-actin	CCTTCCTGGGTATGGAATCCTGT	TAGAGCCACCAATCCACACA
Ppia	Peptidyl-prolyl cis-trans isomerase A	AGCACTGGGGAGAAAGGATT	AGCCACTCAGTCTTGGCAGT
Gapdh	Glyceraldehyde-3-phosphate	GGGTGTGAACCACGAGAAAT	ACTGTGGTCATGAGCCCTTC
Ywhaz	Tyrosine 3-monooxygenase/tryptophan 5-monooxygenase activation protein, zeta	TTGAGCAGAAGACGGAAGGT	GAAGCATTGGGGATCAAGAA
**Myelin-related genes**			
Cldn11	Claudin 11	CGCAAAATGGACGAACTGGG	TGCACGTAACCAGGGAGGAT
Mag	Myelin-associated glycoprotein	AAGCCAGACCATCCAACCTTC	CTCCTGATTCCGCTCCAAGT
Mbp	Myelin basic protein	CCCTACTCCATCCTCAGACTTTCTT	TGGCGGTGTGCCTGTCTAT
Mobp	Myelin-associated oligodendrocytebasic protein	AATACCTGCAGGGCAACAAAG	TCTGGTTCTTGGAGAGCCTGG
Mog	Myelin oligodendrocyte glycoprotein	CGCCGTGGAGTTGAAAGTAG	GCACGGAGTTTTCCTCTAGT
Opalin	Oligodendrocytic Myelin Paranodal And Inner Loop Protein	ACCCTGATCCAGCGAAGAAG	TGACTGCCTAGGATTCTCGGATA
Plp1	Proteolipid protein 1	GGGCCTGAGCGCAACGGTAA	CAGGCACAGCAGAGCAGGCAA
**Glutathione-related genes**			
Gsta4	Glutathione-S-transferase alpha4	GCCGCCAAGTACAACTTGTA	CACTGCTAAAGCTAGGCTCTCTTCTT
Gstm4	Glutathione-S-transferase mu 4	GCCTAGGCCCCTGGTTTTC	TCTTCACAGCAGCACAGCAACT
Prdx6	Peroxiredoxin 6	TGACTGGAAGAAGGGAGAGAGTGT	ATGGGAGCTCTTTGGTGAAGAC
Gclc	Glutamate—cysteine ligase catalytic subunit	AGAGGACAAACCCCAACCAC	TCGTGCAAAGAGCCTGATGT
Gss	Glutathione synthetase	AGCGTGCCATAGAGAACGAG	GCTTCCCAGTTCTGTGCGTT
Gstm1	Glutathione-S-transferase mu 1	GTCATGCCCACATAGTCTTCATTC	AGTTCAGGGCAGACCTCAAATC

Gstm6	Glutathione-S-transferase mu 6	CTGAGCGTTGCTATCTCGGAG	TTCTGTGTATTCCAGGAGCAGC
Anpep	Alanyl Aminopeptidase	CCCATCAGTGGTTTGGCAAC	CATAGTCAGCACCCAGAAATTCC

Supplementary Table 2 – Statistical values of Independent samples T-test with Benjamini Hochberg multiple comparisons correction on qPCR expression data presented in Figure 1.

Age	Gene	t-value	df	p-value	n-number before outlier exclusion	n-number after outlier exclusion
P0	gstm1	-3.184	19	0.005*	APO-SUS n=12<br>APO-UNSUS n=9	No significant outliers
	gstm4	4.841	19	0.000*	APO-SUS n=12<br>APO-UNSUS n=9	No significant outliers
	gsta4	3.652	18	0.002*	APO-SUS n=12<br>APO-UNSUS n=8	No significant outliers
	prdx6	-1.402	19	0.177	APO-SUS n=12<br>APO-UNSUS n=9	No significant outliers
	gstm6	-3.468	19	0.003*	APO-SUS n=12<br>APO-UNSUS n=9	No significant outliers
	anpep	-3.070	8.378	0.015*	APO-SUS n=12<br>APO-UNSUS n=9	No significant outliers
P7	gstm1	-3.184	19	0.005*	APO-SUS n=11<br>APO-UNSUS n=11	No significant outliers
	gstm4	5.494	15.065	0.000*	APO-SUS n=11<br>APO-UNSUS n=12	No significant outliers
	gsta4	7.191	20	0.000*	APO-SUS n=11<br>APO-UNSUS n=11	No significant outliers
	prdx6	-3.100	20	0.006*	APO-SUS n=10<br>APO-UNSUS n=12	No significant outliers
	gstm6	-8.665	21	0.000*	APO-SUS n=11<br>APO-UNSUS n=12	No significant outliers
	anpep	-3.979	20	0.001*	APO-SUS n=11<br>APO-UNSUS n=11	No significant outliers
P14	gstm1	-2.296	20	0.033*	APO-SUS n=12<br>APO-UNSUS n=12	No significant outliers
	gstm4	10.977	22	0.000*	APO-SUS n=12<br>APO-UNSUS n=12	No significant outliers
	gsta4	8.099	20	0.000*	APO-SUS n=11<br>APO-UNSUS n=11	No significant outliers
	prdx6	-8.349	22	0.000*	APO-SUS n=12<br>APO-UNSUS n=12	No significant outliers
	gstm6	-8.912	22	0.000*	APO-SUS n=12<br>APO-UNSUS n=12	No significant outliers
	anpep	-2.374	22	0.027*	APO-SUS n=12<br>APO-UNSUS n=12	No significant outliers

**P21**	gstm1	-4.385	22	0.000*	APO-SUS n=12<br>APO-UNSUS n=12	No significant outliers
	gstm4	6.433	20	0.000*	APO-SUS n=10<br>APO-UNSUS n=12	No significant outliers
	gsta4	8.913	18	0.000*	APO-SUS n=9<br>APO-UNSUS n=11	No significant outliers
	prdx6	-6.683	16.914	0.000*	APO-SUS n=10<br>APO-UNSUS n=12	No significant outliers
	gstm6	-4.280	20	0.000*	APO-SUS n=10<br>APO-UNSUS n=11	No significant outliers
	anpep	-2.784	19	0.012*	APO-SUS n=10<br>APO-UNSUS n=11	No significant outliers
**P90**	gstm1	-3.829	16.795	0.001*	APO-SUS n=10<br>APO-UNSUS n=11	No significant outliers
	gstm4	4.446	5.128	0.006*	APO-SUS n=6<br>APO-UNSUS n=6	APO-SUS n=4<br>APO-UNSUS n=6
	gsta4	2.526	10	0.030*	APO-SUS n=6<br>APO-UNSUS n=6	No significant outliers
	prdx6	-3.513	10	0.006*	APO-SUS n=6<br>APO-UNSUS n=6	No significant outliers
	gstm6	-3.111	10	0.011*	APO-SUS n=6<br>APO-UNSUS n=6	APO-SUS n=5<br>APO-UNSUS n=6
	anpep	-1.783	10	0.105	APO-SUS n=6<br>APO-UNSUS n=6	APO-SUS n=6<br>APO-UNSUS n=5
**P365**	gstm1	-3.760	10	0.004*	APO-SUS n=6<br>APO-UNSUS n=6	APO-SUS n=6<br>APO-UNSUS n=5
	gstm4	3.921	14	0.002*	APO-SUS n=8<br>APO-UNSUS n=8	No significant outliers
	gsta4	2.399	14	0.031*	APO-SUS n=8<br>APO-UNSUS n=8	No significant outliers
	prdx6	-3.719	14	0.002*	APO-SUS n=8<br>APO-UNSUS n=8	No significant outliers
	gstm6	-1.673	14	0.117	APO-SUS n=8<br>APO-UNSUS n=8	No significant outliers
	anpep	-1.170	14	0.261	APO-SUS n=8<br>APO-UNSUS n=8	No significant outliers
	gstm1	0.684	14	0.505	APO-SUS n=8<br>APO-UNSUS n=8	No significant outliers

* Significant

Supplementary Table 3 – Statistical values of Independent samples T-test with Benjamini Hochberg multiple comparisons correction of qPCR expression data presented in Figure 2.

	APO-SUS versus APO-UNSUS	APO-SUS versus APO-SUS+NAC	n-number before	n-number after

Gene	t-value	df	p-value	t-value	df	p-value	outlier exclusion	outlier exclusion
**Prdx6**	-3.157	15	0.007*	0.021	16	0.983	APO-SUS n=9 APO-SUS + NAC n=9 APO-UNSUS n=8 APO-UNSUS + NAC n=8	No significant outliers
**Gstm4**	1.115	10.736	0.289	-0.197	11.573	0.847	APO-SUS n=9 APO-SUS + NAC n=9 APO-UNSUS n=8 APO-UNSUS + NAC n=8	No significant outliers
**Anpep**	-2.362	14	0.033*	-3.070	16	0.007*	APO-SUS n=9 APO-SUS + NAC n=9 APO-UNSUS n=8 APO-UNSUS + NAC n=8	APO-SUS n=9 APO-SUS + NAC n=9 APO-UNSUS n=7 APO-UNSUS + NAC n=8
**Gstm6**	-2.837	6.303	0.028*	-3.139	8.533	0.013*	APO-SUS n=8 APO-SUS + NAC n=9 APO-UNSUS n=7 APO-UNSUS + NAC n=8	APO-SUS n=8 APO-SUS + NAC n=9 APO-UNSUS n=6 APO-UNSUS + NAC n=8
**Gsta4**	-1.190	14	0.254	-6.897	15	0.000*	APO-SUS n=9 APO-SUS + NAC n=9 APO-UNSUS n=8 APO-UNSUS + NAC n=8	APO-SUS n=9 APO-SUS + NAC n=9 APO-UNSUS n=7 APO-UNSUS + NAC n=8
**Gstm1**	-2.568	14	0.022*	-5.476	15	0.000*	APO-SUS n=9 APO-SUS + NAC n=9 APO-UNSUS n=8 APO-UNSUS + NAC n=8	APO-SUS n=9 APO-SUS + NAC n=8 APO-UNSUS n=7 APO-UNSUS + NAC n=8
**Gclc**	-1.782	14	0.096	-4.570	15	0.000*	APO-SUS n=9 APO-SUS + NAC n=9 APO-UNSUS n=8 APO-UNSUS + NAC n=8	APO-SUS n=9 APO-SUS + NAC n=9 APO-UNSUS n=7 APO-UNSUS + NAC n=8
**Gss**	-2.415	7.096	0.046*	-3.013	9.440	0.014*	APO-SUS n=9 APO-SUS + NAC n=9	No significant outliers

							APO-UNSUS n=7<br>APO-UNSUS + NAC n=8	
**Gstm7**	-3.175	13	0.007*	-6.484	13	0.000*	APO-SUS n=8<br>APO-SUS + NAC n=8<br>APO-UNSUS n=7<br>APO-UNSUS + NAC n=8	APO-SUS n=8<br>APO-SUS + NAC n=7<br>APO-UNSUS n=7<br>APO-UNSUS + NAC n=8

* Significant

Supplementary Table 4 – Statistical values of Independent samples T-test with Benjamini Hochberg multiple comparisons correction of qPCR expression data presented in Figure 3.

	APO-SUS versus APO-UNSUS			APO-SUS versus APO-SUS+NAC			n-number before outlier exclusion	n-number after outlier exclusion
Gene	t-value	df	p-value	t-value	df	p-value		
**Mog**	-3.719	15	0.002*	-6.022	15	0.002*	APO-SUS n=9<br>APO-SUS + NAC n=9<br>APO-UNSUS n=8<br>APO-UNSUS + NAC n=8	APO-SUS n=9<br>APO-SUS + NAC n=8<br>APO-UNSUS n=8<br>APO-UNSUS + NAC n=8
**Plp**	-2.495	14	0.026*	-4.769	15	0.026*	APO-SUS n=9<br>APO-SUS + NAC n=9<br>APO-UNSUS n=7<br>APO-UNSUS + NAC n=8	APO-SUS n=9<br>APO-SUS + NAC n=8<br>APO-UNSUS n=7<br>APO-UNSUS + NAC n=8
**Mag**	-2.901	13	0.012*	-4.253	15	0.012*	APO-SUS n=9<br>APO-SUS + NAC n=9<br>APO-UNSUS n=8<br>APO-UNSUS + NAC n=8	APO-SUS n=9<br>APO-SUS + NAC n=8<br>APO-UNSUS n=7<br>APO-UNSUS + NAC n=8
**Mobp**	-2.929	14	0.011*	-2.942	14	0.011*	APO-SUS n=8<br>APO-SUS + NAC n=8<br>APO-UNSUS n=8<br>APO-UNSUS + NAC n=8	No significant outliers
**Cldn11**	-3.740	13	0.002*	-4.424	14	0.002*	APO-SUS n=8<br>APO-SUS + NAC n=8<br>APO-UNSUS n=7<br>APO-UNSUS + NAC n=8	No significant outliers
**Mbp**	-2.162	14	0.048*	-3.010	16	0.048*	APO-SUS n=9<br>APO-SUS + NAC n=9<br>APO-UNSUS n=8	APO-SUS n=9<br>APO-SUS + NAC n=0<br>APO-UNSUS n=7

							APO-UNSUS + NAC n=8	APO-UNSUS + NAC n=8
**Opalin**	-4.025	13	0.001*	-4.667	15	0.001*	APO-SUS n=9<br>APO-SUS + NAC n=9<br>APO-UNSUS n=7<br>APO-UNSUS + NAC n=8	APO-SUS n=8<br>APO-SUS + NAC n=9<br>APO-UNSUS n=8<br>APO-UNSUS + NAC n=8

* Significant

Supplementary Table 5 – Ingenuity pathway analysis (IPA) of RNA-sequencing data from medial prefrontal cortex (mPFC) of APO-SUS versus APO-UNSUS rats.

Ingenuity Canonical Pathways	-log(B-H p-value)	Ratio
**Glutathione-mediated Detoxification**	2.69	0.258
**Glutathione Redox Reactions I**	2.02	0.25
**Complement System**	1.87	0.189
**Cytotoxic T Lymphocyte-mediated Apoptosis of Target Cells**	1.6	0.188
**Calcium-induced T Lymphocyte Apoptosis**	2.56	0.167

Supplementary Table 6 - Genes in Ingenuity pathway analysis (IPA) canonical pathway ‘Glutathione-mediated detoxification’. Transcriptome-wide analysis of APO-SUS and APO-UNSUS medial prefrontal cortex (mPFC) combined with IPA revealed that this pathway represents the most significantly enriched pathway.

Symbol	Entrez Gene Name	Ratio	Corrected p-value
**Gsta1**	glutathione S-transferase alpha 1	1.95	0.0264
**GSTA1**	glutathione S-transferase alpha 1	-2.01	0.00111
**Gsta4**	glutathione S-transferase, alpha 4	2.02	0.00025
**GSTM4**	glutathione S-transferase mu 4	1.2	0.0354
**GSTM5**	glutathione S-transferase mu 5	-1.21	0.000000969
**Gstt1**	glutathione S-transferase, theta 1	-1.65	0.00713
**HPGDS**	hematopoietic prostaglandin D synthase	10.42	0.000794
**MGST1**	microsomal glutathione S-transferase 1	1.46	0.00282

Supplementary Table 7 - Genes in Ingenuity pathway analysis (IPA) canonical pathway ‘Glutathione redox reactions I’. Transcriptome-wide analysis of APO-SUS and APO-UNSUS medial prefrontal cortex (mPFC) combined with IPA revealed that this pathway represents the second-most significantly enriched pathway.

Symbol	Entrez Gene Name	Ratio	Corrected p-value
**GPX3**	glutathione peroxidase 3	-1.2	0.0183
**Gsta1**	glutathione S-transferase alpha 1	1.95	0.0264
**GSTA1**	glutathione S-transferase alpha 1	-2.01	0.00111

**Gstt1**	glutathione S-transferase, theta 1	-1.65	0.00713
**MGST1**	microsomal glutathione S-transferase 1	1.46	0.00282
**PRDX6**	peroxiredoxin 6	-1.64	0.00E+00

Supplementary Table 8 - Top 35 most significantly differentially expressed genes in APO-SUS *versus* APO-UNSUS medial prefrontal cortex (mPFC) as revealed by RNA-sequencing analysis**.**

#	Ratio	Corrected p-value	Symbol	Gene name
**1**	-109.31	0.00E+00	**Igh-1a**	Immunoglobulin heavy chain 1a (serum IgG2a)
**2**	-68.51	0.00E+00	**690499**	rCG64263-like
**3**	-60.45	0.00E+00	**100361105**	Igh protein-like
**4**	12.53	0.00E+00	**Spag6**	Sperm associated antigen 6
**5**	-7.92	0.00E+00	**309627**	RT1 class I, locus A3
**6**	5.81	0.00E+00	**365613**	hypothetical gene supported by X60212
**7**	4.71	0.00E+00	**Vwa3a**	Von Willebrand factor A domain containing 3A
**8**	-4.6	0.00E+00	**Ghdc**	GH3 domain containing
**9**	-3.97	0.00E+00	**Prss12**	Protease, serine, 12 neurotrypsin (motopsin)
**10**	-3.42	0.00E+00	**Acsm3**	Acyl-CoA synthetase medium-chain family member 3
**11**	-3.05	0.00E+00	**LOC362863**	First gene upstream of Nt5dc3
**12**	3	0.00E+00	**RGD1303232**	Phytn_dehydro and Pyr_redox domain containing protein RGD1303232
**13**	-2.85	0.00E+00	**Aph1b**	Anterior pharynx defective 1 homolog B (C. elegans)
**14**	-2.81	0.00E+00	**306478**	ATP synthase, H+ transporting, mitochondrial F0 complex, subunit d-like 1
**15**	-2.69	0.00E+00	**368153**	RT1 class I, locus CE7
**16**	-2.63	0.00E+00	**292539**	similar to 60S ribosomal protein L17 (L23)
**17**	2.49	0.00E+00	**Ifi27l**	Interferon, alpha-inducible protein 27-like
**18**	2.31	0.00E+00	**Zfp61**	Zinc finger protein 61

**19**	-2.28	0.00E+00	**Thumpd2**	THUMP domain containing 2
**20**	2.19	0.00E+00	**Rpl17**	Ribosomal protein L17
**21**	2.02	0.00E+00	**Pla2g4e**	Phospholipase A2, group IVE
**22**	-2	0.00E+00	**Pex11a**	Peroxisomal biogenesis factor 11 alpha
**23**	1.87	0.00E+00	**LOC361963**	Similar to phosphoglycerate mutase (EC 5.4.2.1) B chain – rat
**24**	-1.79	0.00E+00	**Mobp***	Myelin-associated oligodendrocyte basic protein
**25**	-1.79	0.00E+00	**Cables2**	Cdk5 and Abl enzyme substrate 2
**26**	-1.77	0.00E+00	**St7l**	Suppression of tumorigenicity 7-like
**27**	1.75	0.00E+00	**100362124**	intraflagellar transport 140
**28**	-1.72	0.00E+00	**Tmem119**	Transmembrane protein 119
**29**	-1.71	0.00E+00	**RGD735029**	SEL1 domain containing protein RGD735029
**30**	-1.7	0.00E+00	**Opalin***	Oligodendrocytic myelin paranodal and inner loop protein
**31**	-1.69	0.00E+00	**Mbp***	Myelin basic protein
**32**	-1.67	0.00E+00	**Mag***	Myelin-associated glycoprotein
**33**	-1.64	0.00E+00	**Prdx6**	Peroxiredoxin 6
**34**	-1.63	0.00E+00	**Plp1***	Proteolipid protein 1
**35**	-1.59	0.00E+00	**Ptgds**	Prostaglandin D2 synthase (brain)

* Myelin-related genes

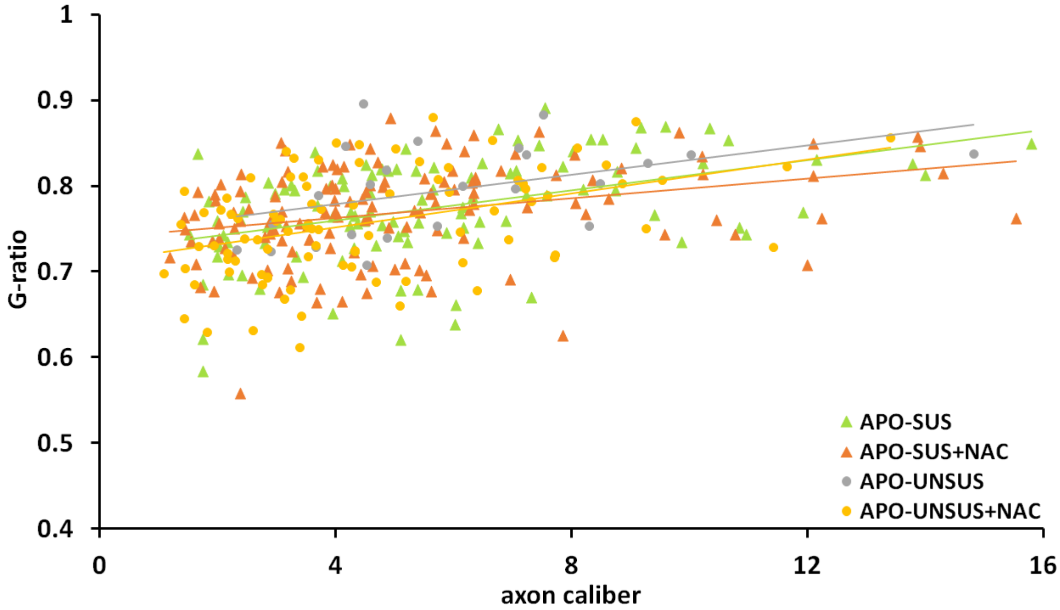

Supplementary Figure S1 – Myelin thickness is not affected in APO-SUS medial prefrontal cortex (mPFC). G-ratio versus axon caliber for all myelinated axons in the infralimbic (IL) subregion of the mPFC of APO-SUS and APO-UNSUS rats with and without NAC treatment.

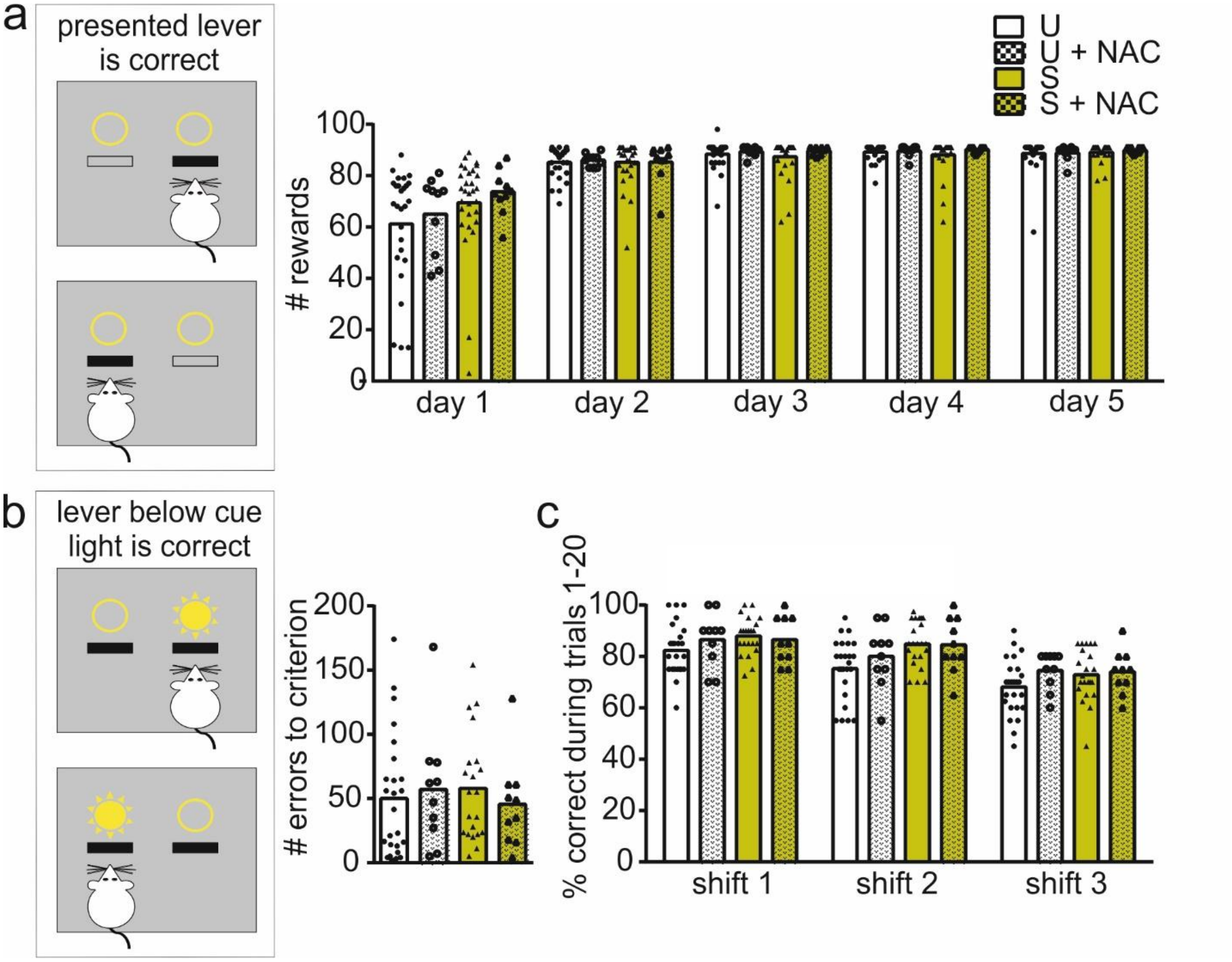

Supplementary Figure S2 – No differences in the performance of APO-SUS and APO-UNSUS rats during retractable lever-press training and visual cue discrimination with and without NAC treatment.

(a) Schematic representation and performance during retractable lever press training. Rats were required to press the lever they were presented with during 5 consecutive sessions, 1 session per day. The number of rewards in each session involving APO-SUS versus APO-UNSUS rats with and without NAC treatment is depicted. **(b)** Schematic representation and quantification of performance during the acquisition of the initial rule. Rats were required to press the lever above which a cue light was illuminated in order to receive a reward pellet. The number of errors until criterion of a streak of 10 correct trials was reached for APO-SUS and APO-UNSUS rats with and without NAC treatment is depicted (Two-way ANOVA interaction $F[1,77]= 0.603$, $p=0.440$ main effects rat line $F[1,77]= 0.033$, $p=0.857$ treatment $F[1,77]= 0.027$, $p=0.870$). **(c)** Percentage correct trials during the first 20 trials of the extra-dimensional set-shifts in APO-SUS and APO-UNSUS rats with and without NAC treatment. Shift 1 Two-way ANOVA interaction $F[1,77]=0.990$, $p=0.323$ main effects rat line $F[1,77]=0.990$, $p=0.323$ treatment $F[1,77]=0.280$, $p=0.598$; Shift 2 Two-way ANOVA interaction $F[1,77]=1.070$, $p=0.304$ main effects rat line $F[1,77]=6.457$, $p=0.013$ treatment $F[1,77]= 0.489$, $p=0.486$; Shift 3 Two-way ANOVA interaction $F[1,77]=0.718$, $p=0.400$ main effects rat line $F[1,77]=0.439$, $p=0.510$ treatment $F[1,77]=1.792$, $p=0.185$.
